# Emotional Differences in Young and Older Adults: Films as Mood Induction Procedure

**DOI:** 10.3389/fpsyg.2018.01110

**Published:** 2018-07-03

**Authors:** Luz Fernández-Aguilar, Jorge Ricarte, Laura Ros, Jose M. Latorre

**Affiliations:** ^1^Department of Psychology, Faculty of Medicine, University of Castilla-La Mancha, Albacete, Spain; ^2^Research Institute of Neurological Disabilities, University of Castilla-La Mancha, Albacete, Spain

**Keywords:** mood induction, emotion response, emotion regulation, film clips, aging

## Abstract

Film clips are proven to be one of the most efficient techniques in emotional induction. However, there is scant literature on the effect of this procedure in older adults and, specifically, the effect of using different positive stimuli. Thus, the aim of the present study was to examine emotional differences between young and older adults and to know how a set of film clips works as mood induction procedure in older adults, especially, when trying to elicit attachment-related emotions. To this end, we use this procedure to analyze differences in subjective emotional response between young and older adults. A sample of 57 older adults and 83 young adults watched a film set previously validated in young population. Their responses were studied in an individual laboratory session to elicit 6 target emotions (disgust, fear, sadness, anger, amusement and tenderness) and neutral state. Self-reported emotional experience was measured using the Self-Assessment Manikin (SAM). Our results show that film clips are capable of evoking positive and negative emotions in older adults. Furthermore, older adults experienced more intensely negative emotions than young adults, especially in response to disgust and fear clips. They also reported higher arousal than young adults, especially in the case of sadness, anger and tenderness clips. Nevertheless, the older adults recovered more easily from the effects of the emotion induction. The young adults reported higher arousal ratings than older adults in response to amusement film clips. On the other hand, this study reflects the importance of controlling the baseline state to study the real strength of mood induction. Overall, current data suggests significant differences occur in emotional response in adult age and that film clips are an effective tool for studying positive and negative emotions in aging research.

## Introduction

It is important to understand the differences between young and older adults in emotional states and reaction. Many of the theoretical models studying emotional experience across adulthood predict changes throughout this life stage. A growing number of studies find that, as we age, the way we understand, manage, and react to positive and negative events changes. Different theoretical models have been proposed to explain this phenomenon: (a) Socioemotional Selectivity Theory; (b) Strength And Vulnerability Integration; and (c) Dynamic integration theory.

One of the most widely espoused theories in recent years is the Socioemotional Selectivity Theory (SST). The SST maintains that time horizons play a key role in motivation (Carstensen, 2006). The future time perspective considers that when the subjective sense of time and its limits changes, our motivational priorities also shift. The theory differentiates two broad categories of goals: one concerning the goals which help us acquire knowledge of the world, and another related to the goals that help us achieve emotional well-being. As people age, they increasingly perceive time as finite. This perception leads older people to prioritize behaviors or goals from which they derive emotional meaning, while younger people prioritize goals related to knowledge acquisition. For example, Hess and his colleagues have shown that older adults, compared to young adults, weighted negative information related to morality more than information regarding competences when judging strangers and rating their likability (Hess, 2005; Leclerc and Hess, 2007). The SST holds that this tendency is even more striking when the categories of goals compete. Moreover, the differences in emotional reactivity do not only manifest in negative emotional states. A recent meta-analysis of 100 independent studies found a reliable positivity effect with older adults showing a positive bias overall and the younger age group showing a negative bias overall (Reed et al., 2014). The “positivity effect” refers to the tendency of older people to prioritize achieving emotional gratification. SST directly connects thinking about a limited future with the emergence of the positivity effect. In short, young adults focused their attention and better remembered negative information while older adults attended to and better remembered positive information (Kennedy et al., 2004). Clearly, individual differences exist. Life events and individuals' management of such variables may positively or negatively impact on the emergence of the positivity effect (Scheibe and Carstensen, 2010).

Strength and Vulnerability Integration (SAVI) is a model associating age-related declines or physiological vulnerabilities with an increase in emotion-regulation strategies (Charles and Luong, 2013). SAVI suggests that in adulthood the functioning of the hypothalamicpituitary-adrenal (HPA) axis and the cardiovascular system diminishes. Activation of these two systems correlates highly with the perception of threat in humans and other species and thus impaired functioning might impact on a subjective decline in negative emotional states. SAVI posits that older adults have self-knowledge about their limited horizon. Then, they are motivated to positive experiences and also the accumulated emotional experience could help them to regulate their emotions. This theory also differentiates between avoidable and unavoidable negative experiences (Charles, 2010). Although elderly are usually oriented and motivated to quickly extricate themselves from negative situations, when negative experiences are highly stressful and inevitable, older adults' recovery is poorer and presents more serious consequences (Charles and Luong, 2013; Piazza et al., 2013).

Dynamic Integration Theory (DIT) relates the decline in cognitive resources to increased vulnerability in situations involving high arousal (Labouvie-Vief, 2003) and a number of studies defend this view. Keil and Freund (2009) showed that in young adults both pleasantness and unpleasantness increased with high emotional arousal, whereas in older adults, low-arousing stimuli were those experienced as most pleasant.

Advances in research and the continued interest in understanding how the emotional system functions in both aging adults and other life stages or life circumstances have generated the development of different Mood Induction Procedures (MIPs). These MIPs can be used to induce positive and negative emotions in a laboratory. Of all the methods implemented thus far, the presentation of film clips with affective content is currently one of the most effective and widely used MIPs (Gerrard-Hesse et al., 1994; Westermann et al., 1996). Film emotion induction is popular for various reasons: (a) simple standardization; (b) high ecological validity; (c) effectiveness in generating responses in the psychophysiological, motor and cognitive systems; (d) capacity to sustain an emotion at both subjective and physiological level for a reasonable time (Carvalho et al., 2012; Jenkins and Andrewes, 2012); and (e) facility to generate discrete emotions (Schaefer et al., 2010). Emotion induction by film clips is especially effective in eliciting negative emotions (Gerrard-Hesse et al., 1994; Westermann et al., 1996; Fernández-Aguilar et al., unpublished). In the literature, there are various published catalogs of film clips for use in research requiring elicitation of different emotions. As emotional targets, these catalogs have examined basic emotions such as anger, fear, disgust, sadness and amusement (Philippot, 1993). Some sets of clips have also included emotions such as surprise and satisfaction (Gross and Levenson, 1995; Rottenberg et al., 2007); tenderness (Schaefer et al., 2010); happiness and mixed emotions (Jenkins and Andrewes, 2012; Samson et al., 2016; Gilman et al., 2017).

Other mood induction procedures have worked successfully to assess emotional reactivity in older adults. For example, the Italian version of the Affective Norms for English Words (ANEW) worked successfully in both healthy aging individuals and Alzheimer's Dementia patients (Mammarella et al., 2017; Di Domenico et al., 2016). However, given the large body of work on film clips as an emotion induction procedure, it is striking that only a few studies have examined the effect of this technique in aging research, and with inconsistent results. Beaudreau et al. (2009) studied the emotional reactions in older adults using the set compiled by Gross and Levenson (1995). They found that older adults reported more anger and less amusement compared to younger adults. The findings of Jenkins and Andrewes (2012) were more generalized. They found that older adults reported higher emotional intensity in response to positive and negative stimuli, especially for clips eliciting fear and amusement. The study by Fajula et al. (2013) revealed similar data but only in the case of negative emotions. Using the set compiled by Philippot (1993), they found that older adults reported higher intensity in the four primary negative emotions (fear, anger, disgust, and sadness) and that young adults reported higher intensity on joy and happiness.

Furthermore, there is a surprising lack of studies on emotion induction addressing other positive emotions apart from the global category of happiness. Attachment-related emotions such as love or tenderness are not usually included. In fact, to date, they have been included in only one database of film clips (Schaefer et al., 2010). Attachment emotions play a significant role in biological, emotional and social development and thus stimuli related to these emotions should be utilized in research on aging. Moreover, different aging models propose a positivity effect whereby older adults are motivated by emotion regulation strategies that maintain positive affective states and by enhanced emotional regulation to recover from negative affect states (Reed et al., 2014). Older adults have been found to favor positive information over negative information in memory and attention (Mather and Carstensen, 2005).

The ambiguity of the previous results motivated us to examine differences in young and older adults as regards their emotional responses when using film clips as the mood induction procedure. This may broaden our knowledge of the characteristics of emotional responses in older adults and how these are explained by models of aging. It also provides the possibility to identify differences between young and older adults in both baseline state and processes of emotional recovery.

Our focus on the baseline state draws on the use of neutral stimuli in a wide range of studies on MIPs. As well as using emotional target stimuli, they also include neutral stimuli in their film sets. Neutral stimuli are used as they enable each participant's' baseline data to be obtained before starting the experimentation and also because they facilitate emotional recovery following the induction of intense emotions. The literature recommends using stimuli free on any type of emotional content and with idiosyncratic characteristics similar to those of the stimuli to be used in the selected MIP (Hewig et al., 2005; Rottenberg et al., 2007). Furthermore, the use of neutral stimuli may help obtain a precise measure of the induction capacity of a specific MIP, considering intraindividually the differences between the state of the participants during exposure to the neutral stimuli and the emotional target stimuli.

The main purpose of this work is to expand our knowledge about fluctuations in positive and negative emotions in older adults when using film clips as a MIP. We compare emotional responses between young and older adults and study the differences between positive and negative induction. To this end, we used clips previously validated in a population of young Spanish adults (see Fernández et al., 2011), the majority of which were elaborated by Schaefer et al. (2010). The following hypotheses were considered: (1) negative mood induction will be more effective compared to positive mood induction both in young and older adults; (2) young and older adults will respond differently to the different negative emotional states induced; (3) young and older adults will respond differently to the different positive emotional states induced; (4) arousal levels will be higher in young adults compared to older adults; (5) baseline state is different in young and older adults and will determine the strength of negative and positive mood induction; and (6) emotion regulation after mood induction will be easier for older adults compared to young adults.

## Materials and methods

### Participants

The final sample comprised 140 volunteers aged between 18 and 84 years (*M* = 39.02, *SD* = 25.32, 68.83% women). From the initial sample, 4 older adults and 7 young adults were excluded due to depressive symptoms. The participants were recruited from a research volunteer pool at the Department of Psychology at the University of Castilla- La Mancha (UCLM) Medical School, from an association at the *Universidad de Mayores* (a university program for older adults) and two socio-cultural centers in the city of Albacete. Participants were divided into age groups to form a younger group of 83 participants aged 18–26 (*M* = 18.87, *SD* = 1.63, 69.9% women) and an older group of 57 participants aged 60–84 years (*M* = 69.74, *SD* = 6.56, 68.4% women). Participants were receiving no psychotropic treatment or drug use and had no previous history of psychological, psychiatric or neurological disorder, according to the criteria of the Diagnostic and Statistical Manual of Mental Disorders Fifth Edition (DSM-V). They presented no auditory or visual impairments other than requiring corrective lenses. All were of Caucasian ethnicity and native Spanish speakers. They gave voluntary consent to take part in the study without obtaining any type of remuneration and according to the requirements of the approved ethics procedure of the Clinical Research Ethics Committee of the Albacete University Hospital.

### Measures

#### Diagnostic evaluation

As depressive symptomatology may affect emotional response, we administered the Beck Depression Inventory II (BDI-II) (Beck et al., 1961) prior to the experiment. The BDI-II is a self-report questionnaire that assesses symptoms of depression including anhedonia, sadness, loss of interest or energy, disturbances in eating and sleeping, loss of concentration or suicidal ideation. On the BDI, scores between 10 and 15 are considered in a dysphoric range and scores of 16 or above represent a depressed range (Kendall et al., 1987). Subjects scoring over 16 were excluded from our study. In the case of the older adults, the Mini Mental State Examination (MMSE) (Folstein et al., 1975) was used to discard cognitive impairment. MMSE is a screening tool measuring symptoms of dementia such as disorientation, alterations in memory, and alterations in the capacity for abstraction or in language. On the MMSE, scores between 9 and 11 are considered in the dementia range, scores between 12 and 24 indicate cognitive impairment, and scores between 24 and 26 suggest suspicion of pathology. Subjects scoring lower than 27 were excluded from our study. Both the BDI-II and the MMSE were administered in a paper-and-pencil version.

The Positive and Negative Affect Schedule- state version (PANAS; Watson et al., 1988) was used to assess positive affect (e.g., interested, excited, proud) and negative affect (e.g., distressed, ashamed, upset) through 20 items with answers ranged between 0 (“not at all”) and 4 (“extremely”). This questionnaire was administered telematically just before starting the experimental session and to assess prior mood before the emotion elicitation procedure.

#### Measurement of emotional response

The subjective emotional response was evaluated using dimensional measures. The Self-Assessment Manikins (SAM) (Bradley and Lang, 1994) is a self-report questionnaire that assesses emotional response, measuring affective valence, arousal and dominance or emotional control. Considering the dimensional structure of affect (Russell and Barrett, 1999), we administered the items measuring valence and arousal. These two dimensions are those most commonly used in the literature (Russell, 1980; Watson et al., 1988) and, furthermore, permit comparison with somato-physiological measures. Thus, participants rated, on a 9-point Likert-type scale, how pleasant/happy/amused (9) or unpleasant/unhappy/sad (1) and how aroused (9) or relaxed (1) they felt while watching the emotional video clips. The questionnaire uses graphic figures which represent the different emotional states and is therefore rapid and simple to administer in both age groups, regardless of participants' educational level.

### Procedure

We selected 54 scenes from HD films dubbed in Spanish with an average length of 2′38″ (see Table 1). These fragments were among those in a battery of audiovisual stimuli validated in a population of young Spanish adults (see Fernández et al., 2011). The selected excerpts maintained the same features used in previous studies (Rottenberg et al., 2007; Schaefer et al., 2010). Furthermore, we added a scene from the film 127 h (Colson et al., 2010) to the disgust category, which presented the characteristics of stimuli used for disgust in previous studies. In accordance with the previously published film clip batteries, each segment was expected to induce an emotion from a specific category: amusement, tenderness, anger, sadness, disgust, fear and neutral state.

**Table 1:** Description of the film clips.

**Film title**	**Length (s)**	**Target emotion**	**Clip description**	**References**
Blue (1)	35	Neutral	Olivier collects papers from a desk Julie	Schaefer et al., 2010
Blue (2)	27	Neutral	leaves the subway and walks through a market	Schaefer et al., 2010
L' amant	47	Neutral	A woman walks through the streets of Saigon and knocks on a door	Schaefer et al., 2010
When Harry met Sally	165	Amusement	Sally simulates an orgasm in a Restaurant	Gross and Levenson, 1995
There's something about Mary (1)	177	Amusement	Ted fights with a dog	Schaefer et al., 2010
There's Something About Mary (2)	144	Amusement	Mary takes sperm from Ted's hair mistaking it for hair gel	Schaefer et al., 2010
The dinner game	102	Amusement	Complex humoristic scene	Schaefer et al., 2010
Dumb and Dumber	93	Amusement	Comic misunderstanding between Lloyd and Harry	Fernández et al., 2011
Benny and Joon	129	Amusement	Benny plays the fool in a coffee shop	Schaefer et al., 2010
Les visiteurs	137	Amusement	Two men wearing medieval armor attack the postman's car	Schaefer et al., 2010
A fish called Wanda	186	Amusement	Archie is found naked by the owners of the house	Schaefer et al., 2010
La vita è bella (2)	108	Tenderness	In a prisoner's camp, a father and a boy talk to the mother using a loud speaker, reaching the whole camp	Schaefer et al., 2010
La vita è bella (3)	260	Tenderness	Mother and son are reunited	Schaefer et al., 2010
La vita è bella (4)	236	Tenderness	In a concentration camp, a parent mistakenly translates the message of an officer so as not to scare his son	Schaefer et al., 2010
Forrest Gump	129	Tenderness	Father and son are reunited	Schaefer et al., 2010
When a Man Loves a Woman	101	Tenderness	Reconciliation between two lovers	Schaefer et al., 2010
Dead Poets Society (2)	271	Tenderness	All the students climb on their desks to express their solidarity with Mr Keating, who has just been fired	Schaefer et al., 2010
Leon	167	Tenderness	Leon and Mathilda are separated forever	Schaefer et al., 2010
E.T	283	Tenderness	E. T. is apparently dying	Schaefer et al., 2010
Ghost	209	Tenderness	The “pottery” scene	Schaefer et al., 2010
Pink Flamingos	31	Disgust	Person eats dog faces	Gross and Levenson, 1995
Trainspotting	105	Disgust	Mark dives into a filthy toilet	Schaefer et al., 2010
Hellraiser	101	Disgust	On the floor, the size of two stains are growing, and progressively transforming into a monster with a human-like skeleton	Schaefer et al., 2010
Seven (3)	206	Disgust	Policemen find the body of a man tied to a table	Schaefer et al., 2010
The dentist	58	Disgust	A man finds a woman whose tongue has been savagely cut off	Schaefer et al., 2010
127 hours	225	Disgust	A man trapped by a rock cuts his own arm	Fernández-Aguilar et al., unpublished
Saving private Ryan	331	Disgust	Graphic war scene: fighting on the beaches	Schaefer et al., 2010
The silence of the lambs(1)	210	Disgust	Forensic examination of a dead body	Schaefer et al., 2010
Schindler's list (2)	115	Anger	Concentration camp commander randomly shoots prisoners from his balcony	Schaefer et al., 2010
Schindler's list (3)	96	Anger	Killing of Jews in a ghetto during WWII	Schaefer et al., 2010
American History X	82	Anger	A neo-Nazi kills an African-American man, smashing his head on the curb	Schaefer et al., 2010
In the name of the father	216	Anger	Violent pólice interrogation leading to forged confessions	Schaefer et al., 2010
Leaving Las Vegas	147	Anger	Sera is raped and beaten by three drunk men	Schaefer et al., 2010
Cry freedom	143	Anger	Police abuse protesters	Gross and Levenson, 1995
Sleepers	146	Anger	Sexual abuse of children	Schaefer et al., 2010
Seven (1)	245	Anger	John Doe tells David that he beheaded his pregnant wife	Schaefer et al., 2010
The piano	48	Anger	Alisdair gets Ada's finger cut off because he is jealous	Schaefer et al., 2010
A perfect world	257	Anger	Butch is gunned down	Schaefer et al., 2010
Scream (2)	225	Fear	A pursuit takes place in a school	Schaefer et al., 2010
The Blair witch project	253	Fear	Final scene in which the characters are apparently killed	Schaefer et al., 2010
The exorcist	100	Fear	A priest tries to cure a girl who is apparently possessed by the devil	Schaefer et al., 2010
The silence of the lambs (2)	200	Fear	Basement chase scene	Gross and Levenson, 1995
Seven (2)	104	Fear	Policemen find the body of a savagely tortured man	Schaefer et al., 2010
The shining	82	Fear	Jack pursues his wife with an axe	Gross and Levenson, 1995
Misery	215	Fear	Annie breaks Paul's legs	Schaefer et al., 2010
Copycat	142	Fear	Helen Hudson gets caught by a murderer in a toilet	Schaefer et al., 2010
City of angels	264	Sadness	Maggie dies in Seth's arms	Schaefer et al., 2010
The Champ	290	Sadness	Boy with dying father	Gross and Levenson, 1995
Schindler's list (1)	78	Sadness	A concentration camp commander randomly shoots prisoners from his balcony	Schaefer et al., 2010
Dangerous minds	138	Sadness	Students in a school class are told that one of their classmates has died	Schaefer et al., 2010
La vita è bella (1)	130	Sadness	In a prisoner's camp, a father and a boy talk to the mother using a loud speaker, reaching the whole camp	Schaefer et al., 2010
Philadelphia	330	Sadness	Andrew and Joe listen opera on the stereo. Andrew describes to Joe the pain and passion felt by the opera character	Schaefer et al., 2010
La vie revée des anges	162	Sadness	Isabelle commits suicide	Schaefer et al., 2010
Dead man walking	414	Sadness	Matthew is put to death by lethal injection	Schaefer et al., 2010
Dead Poets Society (1)	271	Sadness	A schoolboy commits suicide	Schaefer et al., 2010

*References indicate the original studies that validated the use of each film clip*.

The task was programmed and administered using E-prime 2 Professional (Psychology Software Tools, Inc.). Following the recommendations of previous studies (Rottenberg et al., 2007; Fernández et al., 2011), the experiment was conducted in a room with dimmed lighting on a 20″ computer screen situated in a laboratory (8 m^2^). Participants were informed that the aim of the study was to broaden knowledge about emotions. Before starting, participants were asked to be as sincere as possible in their responses and not to think about the answers for too long. It was explained verbally and by means of instructions on the computer screen, that there were no right or wrong answers, and that they should simply express what they felt during the exposure to each of the scenes. They were also told that they were free to drop out at any time they wished. Participants were given basic instructions on how to use the keyboard during the task and how to complete the questionnaire. To facilitate this stage, they were only required to use the numeric keypad and the space bar (marked with a yellow sticker) during the experiment.

In an individual session, the participants started by watching 2 neutral film clips to familiarize themselves with the experimental paradigm and to establish the baseline. Subsequently, they watched 1 film clip for each emotional target (anger, sadness, fear, disgust, tenderness and amusement). To finish the experiment, they watched another neutral film clip to facilitate the process of emotional recovery, particularly following exposure to negative stimuli. The order of the mood induction session was counterbalanced in such a way that (a) participants watched the emotional targets in a different order, (b) two films targeting the same emotion were not shown consecutively, (c) participants did not watch more than two film clips of the same valence consecutively, and (d) neutral film clips were presented in a fixed order before and after the experiment.

**Figure 1:**
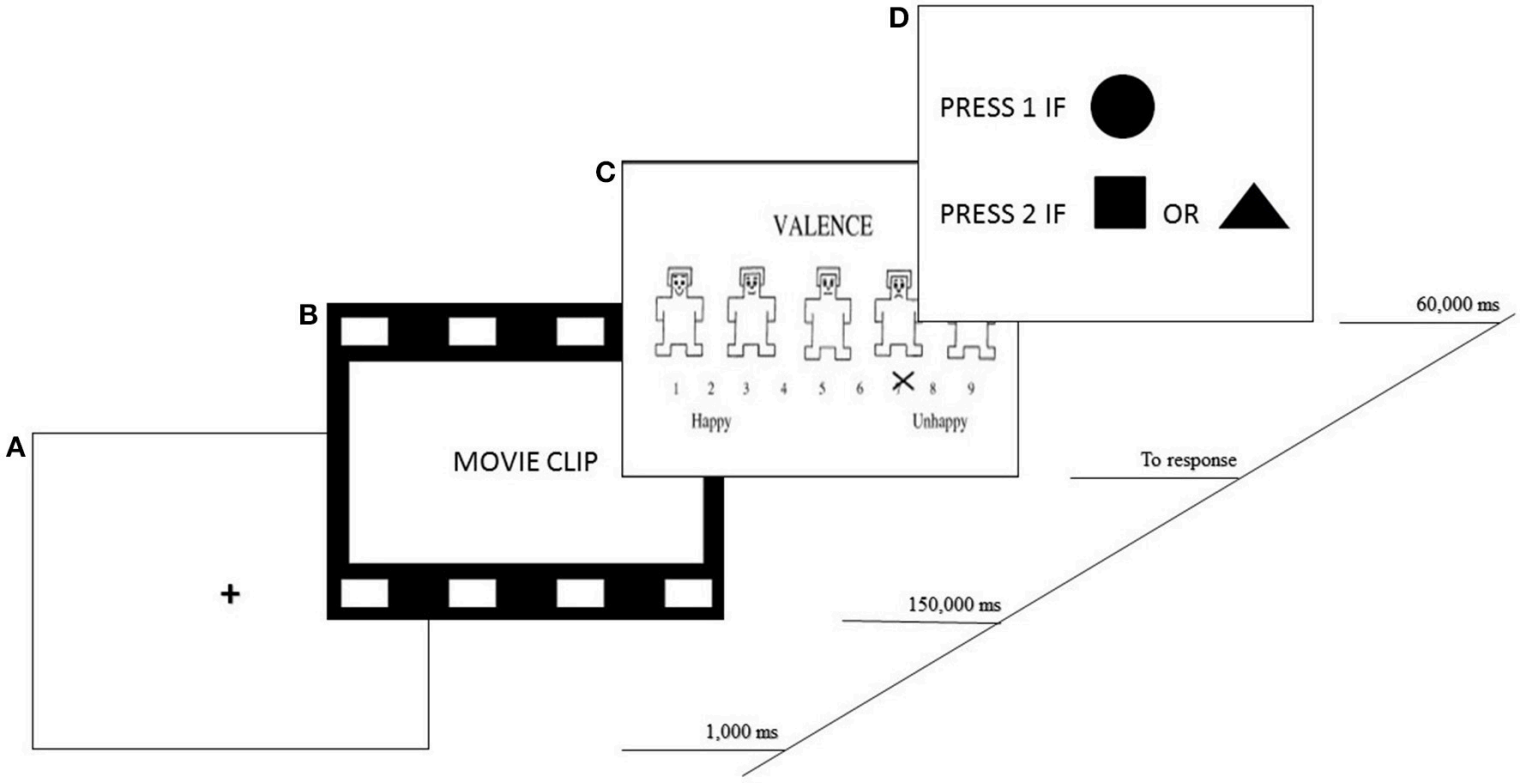
Example of sequence. Time course of events in one sequence. **(A)** Fixation cross. **(B)** Mean length of exposure to the clip. **(C)** Completion of the self-assessment manikin (SAM). **(D)** Distraction task.

Each trial started with a fixation cross in the center of the screen for 500 ms, followed by a neutral, positive or negative film clip. After each of the 9 scenes, participants completed the SAM questionnaire to report their emotional states. The time between clips was approximately 2 min: (a) to complete the questionnaire and (b) to complete a distraction task. The distraction task consisted of tapping the number 2 key when a circle appeared on the screen and the number 1 key when any other shape appeared. Following this, participants pressed the space bar when they felt ready to go on to the next clip. This procedure (including mood induction, emotional self-reports and distraction task) was repeated for each film clip. All tasks were shown on a computer in order not to modify the experimental environment. Figure 1 shows an example of a sequence from one event.

### Statistical procedures

#### Data analysis

Analyses to test our hypotheses were conducted using SPSS 24.0 (IBM) for Windows. The results are organized around 6 basic questions related to age differences: (a) Is negative mood induction by films more effective than positive mood induction in both age groups? (b) Are there differences in negative emotional states between young and older adults? (c) Are there differences in positive emotional states between young and older adults? (d) Are arousal levels higher in young adults compared to older adults? (e) Comparing with baseline scores, are there age differences in the strength of positive and negative induction? (f) Is emotional recovery easier for older adults after mood induction compared to young adults?

#### Preliminary analyses

Our preliminary analyses investigated group differences. As a first step, we examined group differences by age by running descriptive statistics (mean and standard deviation), differences by gender by running a chi-squared test. As a second step, we examined group differences by education level, previous affect state and cognitive state by running an independent samples *t*-test.

#### Mood induction and age

For the main research question of whether emotional states perceived by older adults are different from the emotional states perceived by young people, we conducted separate MANCOVAs to determine the effect of basic emotions and the effect of mood induction procedure by films. Valence and arousal scores were analyzed. Three MANCOVAs were conducted to analyze the negative, positive and neutral categories. Due to the young adults reporting higher negative affect on the PANAS affect scale, this variable was included as a covariate. Furthermore, as the previous literature describes gender-based differences in emotional processing by gender, we also included the gender variable as a covariate. To determine whether the dependent variables were distributed normally within the groups, we conducted the Kolmogorov-Smirnoff test. Independent *t*-tests were conducted to assess the effect of covariates when they were significant. Statistical significance was interpreted by using the criterion of *p* < 0.05.

To identify the real capacity of induction for each emotional category based on the baseline state of each participant, we created six new variables. These variables were derived from the difference in the valence and arousal values for each emotional category and the valence and arousal values reported using the initial neutral film clips. Hence a value was obtained representing this difference for each participant. Three MANCOVAs were conducted to analyze the negative, positive, and neutral categories. PANAS (negative affect) and gender were included as covariates in the analyses. To determine whether the dependent variables were distributed normally within the groups, we conducted the Kolmogorov-Smirnoff test. Independent *t*-tests were conducted to assess the effect of covariates when they were significant. Statistical significance was interpreted by using the criterion of *p* < 0.05.

## Results

Our preliminary analyses revealed differences between groups in some of the variables considered prior to the emotion induction (see Table 2). As regards the affect exhibited by the participants before the experiment, significant differences were observed between the young and older adults, with the young group showing higher negative affect before conducting the task.

**Table 2:** Basic socio-demographic data and participants' scores on prior affect state.

**Variable**	**Young**	**Older**	**Statistical test**	**Cohen's *d***
(n)	83	57		
Age	18.87 (1.69)	69.47 (6.47)		
Gender (%)	69.9	68.4	$\chi_{\left(1\right)}^{2}$ = 0.34, *p* = 0.85	
Female	69.9	68.4		
Male	30.1	31.6		
Education (years)	12.29 (1.04)	12.77 (3.41)	*t*_(138)_ = −1.20, *p* = 0.22	0.19
BDI	7.87 (4.05)	6.68 (4.05)	*t*_(138)_ = 1.69, *p* = 0.09	0.29
MMSE	–	29.21 (0.88)	–	
PANAS +	31.39 (4.92)	30.23 (5.49)	*t*_(138)_ = 1.30, *p* = 0.19	0.22
PANAS −	19.01 (5.13)	14.82 (4.67)	*t*_(138)_ = 4.91, *p* = 0.00	0.85

*Values for young and older adults are M (SD). p > 0.05*.

In relation to the age-related differences in the use of films as an emotion induction procedure, Table 3 presents a general description of the emotional categories, with the associated self-reported ratings of valence and arousal. The ratings are presented separately for young and older adults. The means and standard deviations for each category of film clip were calculated to test the extent to which category scores differed in valence and arousal.

**Table 3:** Valence and arousal of emotional target categories and baseline category.

	**Young (*****n*** = **83)**				**Older (*****n*** = **57)**					
	**Valence**		**Arousal**		**Valence**		**Arousal**		**Valence**	**Arousal**
**Variables**	***M***	***SD***	***M***	***SD***	***M***	***SD***	***M***	***SD***	***p*-value**	***p*-value**
Preinduction baseline	4.95	1.23	3.88	1.66	5.39	1.39	3.03	1.57	0.088	0.026
Disgust	2.35	1.52	5.68	2.06	1.28	0.77	5.91	2.71	0.009	0.213
Fear	2.86	1.68	**7.06**	1.73	1.87	1.40	5.96	2.59	0.017	0.344
Anger	**1.55**	0.77	6.08	2.14	**1.13**	0.44	**6.77**	2.46	0.834	0.002
Sadness	2.83	1.60	5.14	2.00	2.68	2.11	5.51	2.66	0.364	0.025
Amusement	6.12	2.24	4.49	2.21	5.65	2.84	3.60	2.45	0.034	0.931
Tenderness	**6.81**	2.18	4.90	2.14	**6.05**	2.90	5.53	2.61	0.093	0.000
Postindution baseline	4.39	1.30	**2.70**	1.54	4.74	1.18	**2.94**	1.75	0.039	0.416

*Subject-level means and standard deviations of baseline, negative and positive induction categories. Mean values in bold indicate the highest and lowest mean value in emotional film categories for each group. p value refers to the results taking into account the baseline*.

In the assessment of negative emotions, an 8 (valence and arousal: anger vs. fear vs. sadness vs. disgust) × 2 (age groups: young vs. older adults) MANCOVA verified a significant main effect of age group on negative induction, Pillai's trace, [*V* = 0.32, *F*_(8, 107)_ = 6.30, *p* ≤ 0.001].Specifically, the older adults reported significantly higher negative affect in the conditions of disgust, [*F*_(1, 114)_ = 18.18, *p* ≤ 0.001, partial η^2^ = 0.13]; fear, [*F*_(1, 114)_ = 16.90, *p* ≤ 0.001, partial η^2^ = 0.12], and anger, [*F*_(1, 114)_ = 15.05, *p* ≤ 0.001 partial η^2^ = 0.11]. The young adults exhibited significantly higher levels of arousal in the fear condition, [*F*_(1, 114)_ = 4.49, *p* = 0.036, partial η^2^ = 0.03]. PANAS covariate (negative affect) remained significant in valence, Pillai's trace, [*V* = 0.11, *F*_(8, 107)_ = 1.70, *p* = 0.104, partial η^2^ = 0.11]. Specifically, in the fear condition, [*F*_(1, 114)_ = 7.07, *p* = 0.009, partial η^2^ = 0.05], [r_xy_ = −0.73, *p* = 0.39], and the anger condition, [*F*_(1, 114)_ = 4.18, *p* = 0.043, partial η^2^ = 0.03], [r_xy_ = −0.09, *p* = 0.28].

Gender covariate remained significant in the valence dimension, Pillai's trace, [*V* = 0.07, *F*_(8, 107)_ = 1.07, *p* = 0.389, partial η^2^ = 0.07]. Specifically in the anger condition, [*F*_(1, 114)_ = 5.96, *p* = .016, partial η^2^ = 0.05]. The independent *t* test revealed that women (*M* = 1.26, *SD* = 0.57) scored significantly lower than men (*M* = 1.67, *SD* = 0.85), [*t*_(120)_ = 2.54, *p* = 0.015] in affective valence on the anger-inducing stimuli.

**Figure 2:**
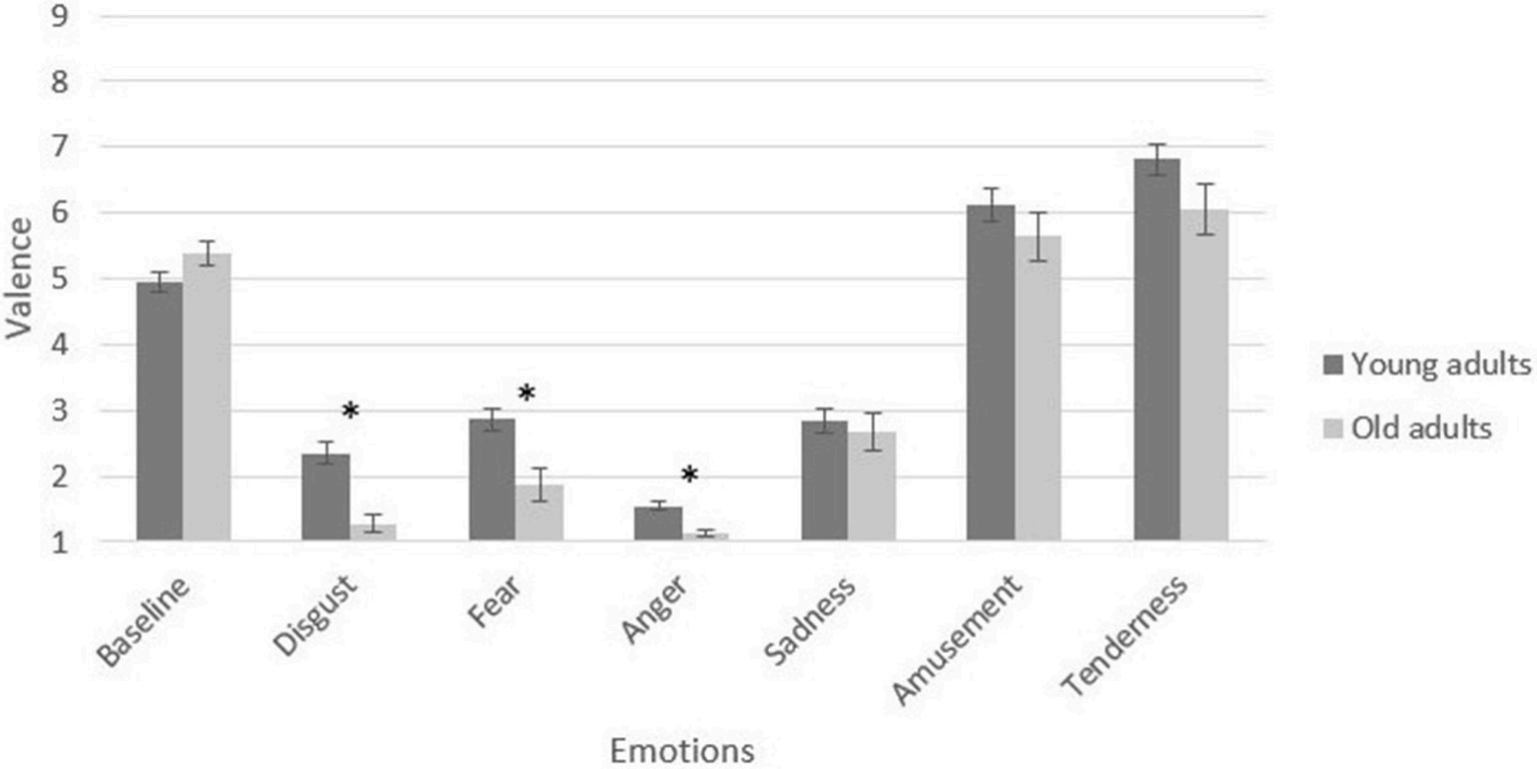
Self-reported valence of participants by film condition and age group. Participant-level means of baseline and emotional target categories. Error bars depict standard error (SE) values. *p < 0.05.

**Figure 3:**
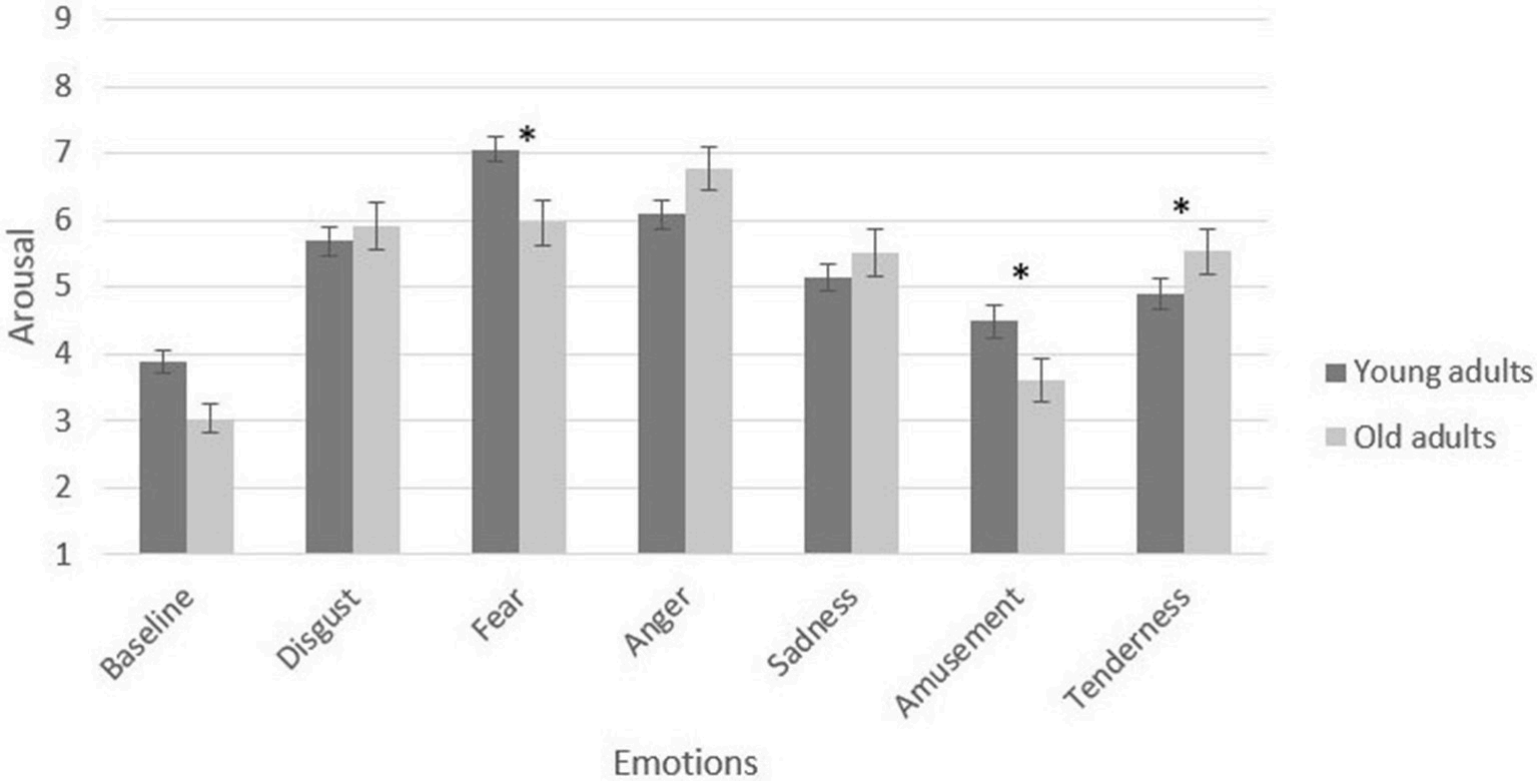
Self-reported arousal of participants by film condition and age group. Participant-level means of baseline and emotional target categories. Error bars depict standard error (SE) values. *p < 0.05.

In the assessment of positive emotions, a 4 (valence and arousal: amusement vs. tenderness) × 2 (age groups: young vs. elderly) MANCOVA verified a significant main effect of age group on positive induction, [*F*_(4, 133)_ = 3.58, *p* = 0.008, partial η^2^ = 0.09]. Specifically, the older adults reported higher arousal in the tenderness condition, [*F*_(1, 136)_ = 3.87, *p* = 0.050, partial η^2^ = 0.02]. The young adults reported higher arousal in the amusement condition, [*F*_(1, 136)_ = 4.28, *p* = 0.040, partial η^2^ = 0.03]. Figures 2, 3 present a graphic description of the effects in valence and arousal of induction in the young and older adult groups.

Our hypothesis was that taking into account the baseline state of each participant would reveal differences in the capacity of induction of positive and negative emotions in young and older adults. Thus, an 8 (valence and arousal: anger vs. fear vs. sadness vs. disgust) × 2 (age groups: young vs. older adults) MANCOVA verified a significant main effect of age group on negative induction when considering the baseline measure for each participant, Pillai's trace, [*V* = 0.19, *F*_(8, 129)_ = 3.89, *p* ≤ 0.001, partial η^2^ = 0.19]. On comparison with baseline states, older adults revealed more substantial differences in valence for the conditions of disgust, [*F*_(1, 136)_ = 6.98, *p* = 0.009, partial 0.04], and fear, [*F*_(1, 136)_ = 5.83, *p* = 0.017, partial η^2^ = 0.04]. The older adults, on comparison with baseline states, also showed more substantial changes in arousal for the conditions of anger [*F*_(1, 136)_ = 10.02, *p* = 0.002, partial η^2^ = 0.06], and sadness [*F*_(1, 136)_ = 5.15, *p* = 0.025, partial η^2^ = 0.03]. Moreover, gender covariate remained significant in arousal, Pillai's trace [*V* = 0.06, *F*_(8, 129)_ = 1.13, *p* = 0.346, partial η^2^ = 0.06], in the anger condition [*F*_(1, 136)_ = 4.97, *p* = 0.027, partial η^2^ = 0.03]. The women (*M* = 3.09, *SD* = 2.61) scored significantly higher than men (*M* = 2.05, *SD* = 2.51) on arousal generated by anger-eliciting stimuli, *t*_(138)_ = −2.19, *p* = 0.030.

**Figure 4:**
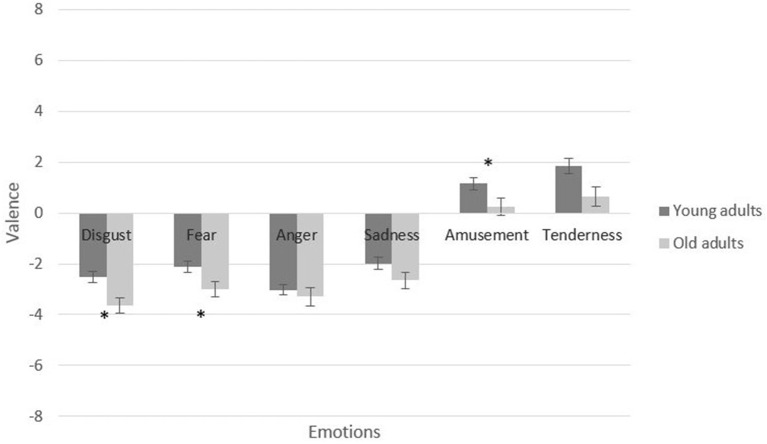
Self-reported valence of participants by film condition and age group. Participant-level means of differences between targets scores and baseline categories. Error bars depict standard error (SE) values. *p < 0.05.

**Figure 5:**
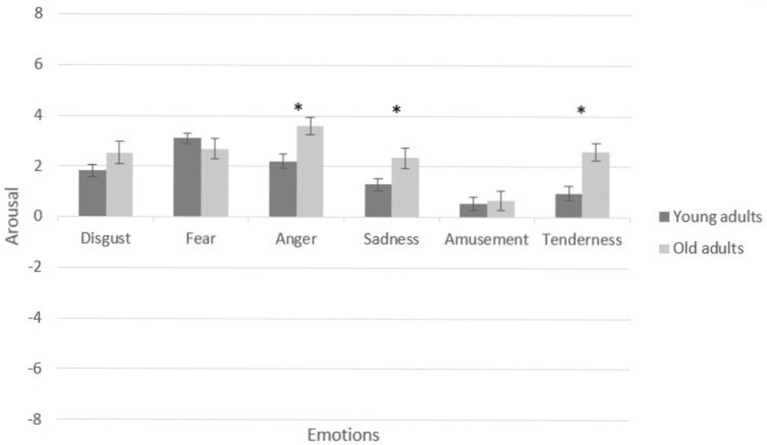
Self-reported arousal of participants by film condition and age group. Participant-level means of differences between emotional targets scores and baseline categories. Error bars depict standard error (SE) values. *p < 0.05.

Furthermore, a 4 (valence and arousal: amusement vs. tenderness) × 2 (age groups: young vs. older adults) MANCOVA verified a significant main effect of age group on positive induction when considering the baseline measure of each participant, [*F*_(4, 133)_ = 5.95, *p* ≤ 0.001, partial η^2^ = 0.15]. Specifically, the young adults showed a more substantial difference between the valence reported at the baseline measurement and that evoked by the amusement condition, [*F*_(1, 136)_ = 4.60, *p* = 0.034, partial η^2^ = 0.03]. In the tenderness condition, the older adults showed a greater difference between their baseline level of arousal and the tenderness condition, [*F*_(1, 136)_ = 12.79, *p* ≤ 0.001, partial η^2^ = 0.08]. Figures 4, 5 present a graphic description of the effects in valence and arousal of induction in the young and older adults when baseline state is considered.

Finally, we hypothesized that the older adults would recover more easily from the effects of the emotion induction. Confirming this hypothesis, a 4 (valence and arousal: neutral state before the task vs. neutral state after the task) × 2 (age groups: young vs. older adults) MANCOVA verified a significant main effect of age group on neutral induction, [*F*_(4, 116)_ = 2.70, *p* = 0.034, partial η^2^ = 0.08]. Specifically, the young adults reported a higher level of arousal above their baseline state compared to the older adults [*F*_(1, 119)_ = 5.11, *p* = 0.026, partial η^2^ = 0.04]. As regards recovery after the different inductions, the older adults reported higher levels of valence than the young adults [*F*_(1, 119)_ = 4.33, *p* = 0.039, partial η^2^ = 0.03].

Regarding the power of this study, given a total sample size of *N* = 140, α = 0.05, two groups and the number of variables introduced in the model, and minimum global effects of size *f* = 0.48, the minimum *post hoc* power value obtained in this study was 0.99. Power calculations were conducted using G^*^Power (Faul et al., 2007).

## Discussion

Using film clips to elicit different positive and negative emotions, the main aim of the present study was to analyze whether young and older adults exhibit differences in their emotional responses. We included emotional video clips from previous studies (Rottenberg et al., 2007; Schaefer et al., 2010; Fernández et al., 2011) which had successfully induced different emotional states. The set of film clips were selected to cover the four subquadrants of the affective space, based on the multidimensional model of emotion: valence and arousal in bimodal lines from high to low. To compare emotional response, clips were classified in seven emotional categories, two were positive emotions (amusement and tenderness), four were negatives emotions (fear, anger, sadness, and disgust), and one was classified as neutral. The aim of this last category was to establish a baseline measure and to facilitate emotional recovery at the end of the task. Although previous studies have measured baseline with different stimuli, this measure is normally used as a global reference. For example, some studies use neutral stimuli as an experimental condition (e.g., Chou et al., 2007) and others studies establish a specific mean emotion rating on a point scale for each of the target emotions. For example, it is considered a neutral stimulus when the score of this stimulus is a mean emotion rating of less than 2 points on a 10-point scale for each of the target emotions (e.g., Jenkins and Andrewes, 2012). However, the present work uses the neutral baseline measure for each participant to take into account individual differences and to compare the strength of the emotional film set for each participant. We analyzed the positive, negative and neutral emotional induction of the stimuli separately and, the capacity of emotional induction compared with the measure of the baseline state of each participant. In all our analyses, we studied the effect of emotion induction between young and older adults. To discuss the results, we address the questions raised in a previous section:

### (a) Is negative mood induction by films more effective than positive mood induction in both age groups? (b) are there differences in negative emotional states between young and old adults?

The analyses reveal that the strength of emotion elicitation is greater with negative stimulation compared to positive stimulation in both age groups. The response to positive stimuli was lower in both cases. Coinciding with previous studies (e.g., Beaudreau et al., 2009), elicitation of positive emotions appears to be more difficult. Furthermore, age group comparisons revealed that participants aged over 60 years gave higher target ratings on many film clips for negative emotions compared to participants aged under 26 years. In particular, our findings show that older adults reported more affective judgments of unpleasantness than young adults for the audiovisual stimuli of disgust, fear and anger. These results support other previous findings on increased self-reported negative emotions in samples of older adults (e.g., Jenkins and Andrewes, 2012; Fajula et al., 2013). Our review of the literature supports the hypothesis that it is easier to induce negative mood state than positive mood state in a large number of different types of induction procedures (Gerrard-Hesse et al., 1994; Westermann et al., 1996). Furthermore, the literature indicates that older adults respond more intensely to certain stimuli involving scenes of social injustice or a situation of loss or the loss of a loved one (Charles, 2005; Kunzmann and Gruhn, 2005). This might be related to the late adult lifespan and the influential theory of SST, with the existence of a differential susceptibility to emotional information between young and old adults, where older adults attend more to negative information related to moral questions (Leclerc and Hess, 2007). When older adults watched scenes of social or emotional injustice, they reported more unpleasantness because older individuals are more sensitive to socio-emotional information than other types of negative information.

### (c) Are there differences in positive emotional states between young and older adults?

In the case of positive emotions, the current study found no age differences in the valence dimension. Young and older adults show no differences in the levels of pleasantness induced by the stimuli of amusement and tenderness. However, young adults reported more arousal in response to amusement clips compared to older adults, who reported higher arousal in response to tenderness clips than young adults. In the case of amusement, these findings are consistent with those of previous studies comparing emotional response in young and older adults (Beaudreau et al., 2009; Fajula et al., 2013). These differences might be related to changes in the pattern of arousal in young and older adults' emotional responses (Keil and Freund, 2009). However, despite it being a positive emotion, the same response pattern was not found with the stimuli for eliciting tenderness. As previously mentioned, the different systems of emotional induction proposed thus far have neglected attachment-related emotions, and so this question cannot be clearly resolved. In fact, thus far no review of potential discrete positive emotions has been conducted due to the limited number of studies that include more than one category of positive emotions (Bonanno and Keltner, 2004; Lench et al., 2011) However, here it is important to consider that older and young adults differ in the importance they give to meaningful social relationships in which attachment is the core emotion. Young and older adults spend their time on different types of activities. Older adults seem to favor more personally and emotionally meaningful forms of engagement (Hendricks and Cutler, 2004). Investment in meaningful social relationships increases with age while the aging process is associated with selective attention that is especially focused on positive stimuli (Isaacowitz et al., 2006; Charles and Carstensen, 2010). In light of the above, it is unsurprising that individuals experience tenderness-related stimuli more intensely.

### (d) Are arousal levels higher in young adults compared to old adults?

Age group comparisons revealed that the arousal ratings of participants in the young group were higher compared to older adults for all the negative categories. In particular, our findings showed that young adults reported more arousal in response to the fear stimulus. This result is consistent with different aging models such as the Socioemotional Selectivity Theory, Strength and Vulnerability Integration and especially the Dynamic Integration Theory (DIT) because when older people use certain strategies to avoid experiencing negative emotions, they experience higher levels of well-being more easily than do young adults. Moreover, younger adults are more likely to dwell on negative information than older adults (Charles and Carstensen, 2010), which might result in higher levels of arousal. In the case of positive induction, young adults reported more arousal in response to amusement clips compared to older adults, who reported higher arousal in response to tenderness clips than young adults. These results are consistent with the theory of young and older adults responding differently, in terms of arousal, to positive and negative events. Thus, when older adults consider a positive stimulus, they experience a low level of arousal and when they view a stimulus as unpleasant, their arousal is high. For young people, however, in both cases arousal is high (Keil and Freund, 2009). In fact, some studies suggest that high-arousal positive emotions such as euphoria tend to decline with age, while, in contrast, low-arousal emotions such as contentment or feeling at peace increase (Diener et al., 1985; Lawton et al., 1992; Charles and Carstensen, 2010). This might explain why older adults respond to tenderness-inducing clips with higher levels of arousal. If we look at the characteristics of the stimuli used to evoke tenderness, it can be seen that some of the clips involve a situation of conflict. For example, one of the segments is from the film La vita è bella (Ferri and Benigni, 1997). A father and his son are in a concentration camp and the father deliberately mistranslates the orders of a Nazi officer to avoid frightening his son. Another example is the clip from the film Leon (Ledoux and Besson, 1994), in which the two main characters (an adult and a girl) have to say goodbye in the knowledge that the adult character is going to die. These and other segments from this category show situations which reflect personal losses and injustices, and, as mentioned in the previous section, older adults respond more intensely to these types of stimuli. Comparison of the emotional responses of young and older adults reveals that older adults are more sensitive to emotional clues associated with social events (Hess, 2005).

### (e) Comparing with baseline scores, are there age differences in the strength of positive and negative induction?

To determine the real impact of a MIP, it is necessary to compare the effect of the components used with a point of reference. Researchers usually adopt the subjective or physiological score obtained using an emotionally neutral stimulus, or, in other words, a stimulus with no emotional content. In the present study, we selected short segments from films that had already been validated in Spanish population (Fernández et al., 2011). Having analyzed the scores on the initial neutral stimuli, no significant differences were found between young and older adults in valence. However, the older adults exhibited a certain tendency to score the neutral stimuli more positively while the young adults scored them more neutrally. In the case of the arousal dimension, we found age differences in the level of arousal generated by the initial neutral stimuli. In other words, the older adults reacted to the neutral clips in a more relaxed way than the young adults. This is related to the positivity effect in old age and the ability to prioritize the maintenance of emotional equilibrium (Mather and Carstensen, 2005). Indeed, previous findings suggest that older adults exhibit higher levels of pleasantness reactions under low arousal while young adults follow the reverse pattern (Streubel and Kunzmann, 2011). Furthermore, the analysis of the difference in each emotional category respective to each participant's baseline state shows that the results vary, to a certain extent, compared to the analyses of each emotional category in isolation. The effects of the negative induction reveal age differences in valence and arousal. In old age, there is a greater distance between the response to neutral stimuli and stimuli eliciting disgust and fear. As regards the level of activation, the older adults exhibit greater distances between their level of arousal in response to neutral stimuli and in response to stimuli eliciting anger and sadness. This is consistent with the large body of literature supporting the SST, which postulates that older adults show substantial mood changes in a highly salient negative MIP (Carstensen, 1993, 1995; Carstensen and Turk-Charles, 1994), and that these changes are more pronounced when the stimuli used have a particularly high relevance for older adults (i.e., World War II scenes) (Kunzmann and Gruhn, 2005; Kliegel et al., 2007). This is also corroborated by the Strength and Vulnerability Theory (Charles, 2010), which postulates that older adults make strategic use of their resources to avoid the experience of negative affect. This generates higher levels of well-being in older adults compared to young adults. The differences between young and older adults remain significant even when controlling for the contaminating variable of gender. In this sense, it was found that women present higher levels of arousal in response to clips evoking anger. These results are consistent with previous studies reporting that women exhibit a higher subjective arousal level than men in negative affect, and in general more intense negative affect, when viewing film clips (Gross and Levenson, 1995; Hagemann et al., 1999; Schaefer et al., 2010). The effects for positive induction reveal differences between young and older groups in both valence and arousal. There is a greater distance between the baseline state in younger adults compared to older adults in the level of pleasantness generated by amusement clips. In contrast, the older adults exhibit more robust changes in response to clips evoking tenderness.

### (f) Is emotional recovery easier for older adults after mood induction compared to young adults?

After viewing the emotion-eliciting film clips, the participants were exposed to a neutral stimulus, and in the same way as for the prior neutral stimulus, the response was measured. In all the experimental sessions, the last stimulus was neutral in order to facilitate emotional recovery, especially in the case of negative stimuli. Our results show that the older adults exhibit higher levels of valence than young adults with regard to the final neutral stimulus presented. Although the difference is small and non-significant, if we analyze the scores on the SAM scale, which range from 1 to 9, those for the older adults are in the neutral range while those for the young adults are in the unpleasantness range. Our findings might be associated with differences between young and older adults in the process of emotional recovery. In terms of emotion regulation, older adults present fewer difficulties in returning to their baseline state after mood induction (Larcom and Isaacowitz, 2009) and use more effective emotion-regulation strategies to deal with interpersonal tensions (Scheibe and Blanchard-Fields, 2009). In addition, the literature associates components of negative affectivity with ineffective emotion regulation (John and Gross, 2007). In our study, we found that young adults were significantly more negative compared to older adults as measured on the PANAS state version, so these scores might be related. Individuals differ in their use of emotion regulation strategies such as reappraisal and suppression. Various studies suggest that the use of reappraisal is positively related to well-being while suppression relates negatively (Gruber et al., 2014; Nowlan et al., 2015). Thus, it is reasonable to suppose that older adults use reappraisal more than their younger counterparts. However, the literature evidences older adults' preference for using distraction rather than reappraisal. Moreover, reappraisal is a cognitively demanding strategy, involving especially the ventromedial and dorsolateral prefrontal cortex regions. Neuroimaging studies have revealed older adults activate these regions less than younger adults (Weiner, 1982; Mather, 2012, 2016; Allard and Kensinger, 2014; Buhle et al., 2014; Dolcos et al., 2014).

This study also has some limitations that should be mentioned. First, the low participation rate could have given rise to certain issues. For example, some film clips were watched by only a small number of participants. Second, the effectiveness of different MIPs was not compared in the older population. In subsequent studies, it would be advisable to use measures from different MIPs to analyze the capacity of film clips as useful MIP to older adults. In addition, longitudinal studies would be needed to measure changes in emotional response patterns from youth to old age.

## Conclusions and future directions

The aim of this study was to understand the differences between young and older adults in both positive and negative emotions. To this end, a set of film clips was used that would enable us to determine the strength of induction of this procedure in older adults and compare findings with a sample of young adults. A further aim was to reflect on the need to broaden the categories of positive emotions in different systems of emotion elicitation.

Our findings clearly show that film clips evoke differential emotional responses in younger and older adults. The present study also corroborates the importance of measuring the baseline state of each participant using neutral stimuli. Only by considering the baseline state can the strength of the emotion induction be identified since this takes into account the distance between affect at baseline and after induction. In future research, it would be interesting to study the effect of different kinds of film clips in different age groups. Thus, it could be seen whether the effect of using clips which are more, or less, representative of different generations impacts on the capacity of induction. Taking into account the complexity of the emotional system, it would also be interesting to investigate the emotional response across the different channels comprising this system, including the physiological and motor response. Finally, it would be interesting to delve deeper into the underlying reasons for the differences between young and older adults as regards attachment-related emotions and to study the role this might play in the aging process. Emotion induction procedures need to include categories such as tenderness that allow us to enhance knowledge of positive emotions and extend research on these emotions across the lifespan. An exhaustive classification of the film clips used to induce positive emotions is needed. It is also necessary to differentiate the scenes that could possibly provoke a mix of emotions from those that induce an isolated emotion.

## Ethics statement

This study was carried out in accordance with the recommendations of Acta n° 06/2016 of Comité Ético de Investigación Clínica (CEIC). The protocol was approved by the Comité Ético de Investigación Clínica, Servicio de Salud de Castilla La Mancha. All subjects gave written informed consent in accordance with the Declaration of Helsinki.

## Author contributions

LF and JL: Conception and design of the study. LF and LR: Analysis and interpretation of the data. LF and JR: Drafting of the article. LF, JL, LR and JR: Final approval of the article.

### Conflict of interest statement

The authors declare that the research was conducted in the absence of any commercial or financial relationships that could be construed as a potential conflict of interest.

## References

[B1] Allard E. S. Kensinger E. A. (2014). Age-related differences in neural recruitment during the use of cognitive reappraisal and selective attention as emotion regulation strategies. Front. Psychol. 5:296. 10.3389/fpsyg.2014.00296 24782800 PMC3989764

[B2] Beaudreau S. A. MacKay A. Storandt M. (2009). Older adults' responses to emotional stimuli: a cautionary note. Exp. Aging Res. 35, 235–249. 10.1080/03610730902720513 19280449

[B3] BECK A. T. WARD C. H. MENDELSON M. MOCK J. ERBAUGH J. (1961). An inventory for measuring depression. Arch. Gen. Psychiatry 4, 561–571. 10.1001/archpsyc.1961.01710120031004 13688369

[B4] Bonanno G. A. Keltner D. (2004). The coherence of emotion systems: comparing “on-line” measures of appraisal and facial expressions, and self-report. Cogn. Emot. 18, 431– 444. 10.1080/02699930341000149

[B5] Bradley M. M. Lang P. J. (1994). Measuring emotion: the self-assessment manikin and the semantic differential. J. Behav. Ther. Exp. Psychiatry 25, 49–59. 10.1016/0005-7916(94)90063-9 7962581

[B6] Buhle J. T. Silvers J. A. Wager T. D. Lopez R. Onyemekwu C. Kober H.. (2014). Cognitive reappraisal of emotion: a meta-analysis of human neuroimaging studies. Cereb. Cortex 24, 2981–2990. 10.1093/cercor/bht154 23765157 PMC4193464

[B7] Carstensen L. L. (1995). Evidence for a life-span theory of socioemotional selectivity. Curr. Direct. Psychol. Sci. 4, 151–156. 10.1111/1467-8721.ep11512261

[B8] Carstensen L. L. (1993). Motivation for social contact across the life span: a theory of socioemotional selectivity, in Nebraska Symposium on Motivation, ed Jacobs J. E. (Lincoln: University of Nebraska Press), 209–254. 1340521

[B9] Carstensen L. L. (2006). The Influence of a sense of time on human development. Science 312, 1913–1915. 10.1126/science.1127488 16809530 PMC2790864

[B10] Carstensen L. L. Turk-Charles S. (1994). The salience of emotion across the adult life span. Psychol. Aging 9, 259–264. 10.1037/0882-7974.9.2.259 8054174

[B11] Carvalho S. Leite J. Galdo-Álvarez S. Gonçalves O. F. (2012). The emotional movie database (EMDB): a self-report and psychophysiological study. Appl. Psychophysiol. Biofeedback 37, 279–294. 10.1007/s10484-012-9201-6 22767079

[B12] Charles S. T. (2005). Viewing injustice: greater emotion heterogeneity with age. Psychol. Aging 20, 159–164. 10.1037/0882-7974.20.1.159 15769221

[B13] Charles S. T. (2010). Strength and vulnerability integration: a model of emotional well-being across adulthood. Psychology Bulletin, 136, 1068–1091. 10.1037/a0021232 21038939 PMC3059514

[B14] Charles S. T. Carstensen L. L. (2010). Social and emotional aging. Annu. Rev. Psychol. 61, 383–409. 10.1146/annurev.psych.093008.100448 19575618 PMC3950961

[B15] Charles S. T. Luong G. (2013). Emotional experience across adulthood. Curr. Dir. Psychol. Sci. 22, 443–448. 10.1177/0963721413497013

[B16] Chou K.-L. Lee T. M. C. Ho A. H. Y. (2007). Does mood state change risk taking tendency in older adults? Psychol. Aging 22, 310–318. 10.1037/0882-7974.22.2.310 17563186

[B17] Colson S. Smithson J. Boyle D. (2010). 127 Hours. Los Angeles, CA: Fox Searchlight Pictures Pathé.

[B18] Di Domenico A. Palumbo R. Fairfield B. Mammarella N. (2016). Fighting apathy in Alzheimer's dementia: a brief emotional-based intervention. Psychiatry Res. 242, 331–335. 10.1016/j.psychres.2016.06.009 27336799

[B19] Diener E. Sandvik E. Larsen R. J. (1985). Age and sex effects for emotional intensity. Dev. Psychol. 21, 542–546. 10.1037/0012-1649.21.3.542

[B20] Dolcos S. Katsumi Y. Dixon R. A. (2014). The role of arousal in the spontaneous regulation of emotions in healthy aging: a fMRI investigation. Front. Psychol. 5:681. 10.3389/fpsyg.2014.00681 25120498 PMC4112914

[B21] Fajula C. Bonin-Guillaume S. Jouve E. Blin O. (2013). Emotional reactivity assessment of healthy elderly with an emotion-induction procedure. Exp. Aging Res. 39, 109–124. 10.1080/0361073x.2013.741961 23316739

[B22] Faul F. Erdfelder E. Lang A. G. Buchner A. (2007). G^*^Power 3: a flexible statistical power analysis program for the social, behavioral, and biomedical sciences. Behav. Res. Methods 39, 175–191. 10.3758/BF03193146 17695343

[B23] Fernández C. F. Mateos J. C. P. Ribaudi J. S. Fernández-Abascal E. G. (2011). Spanish validation of an emotion-eliciting set of films. Psicothema, 23, 778–785. 22047873

[B24] Ferri E. Benigni R. (1997). La Vita è Bella. Rome: Melampo Cinematografica.

[B25] Folstein M. F. Folstein S. E. McHugh P. R. (1975). “Mini-mental state.” A practical method for grading the cognitive state of patients for the clinician. J. Psychiatr. Res. 12, 189–198. 10.1016/0022-3956(75)90026-6 1202204

[B26] Gerrard-Hesse A. Spies K. Hesse F. W. (1994). Experimental inductions of emotional states and their effectiveness: a review. Br. J. Psychol. 85, 55–78. 10.1111/j.2044-8295.1994.tb02508.x

[B27] Gilman T. L. Shaheen R. Nylocks K. M. Halachoff D. Chapman J. Flynn J. J.. (2017). A film set for the elicitation of emotion in research: a comprehensive catalog derived from four decades of investigation. Behav. Res. Methods 49, 2061–2082. 10.3758/s13428-016-0842-x 28078572

[B28] Gross J. J. Levenson R. W. (1995). Emotion elicitation using films. Cogn. Emot. 9, 87–108. 10.1080/02699939508408966

[B29] Gruber J. Hay A. C. Gross J. J. (2014). Rethinking emotion: cognitive reappraisal is an effective positive and negative emotion regulation strategy in bipolar disorder. Emotion 14, 388–396. 10.1037/a0035249 24364852

[B30] Hagemann D. Naumann E. Maier S. Becker G. Lürken A. Bartussek D. (1999). The assessment of affective reactivity using films: validity, reliability and sex differences. Pers. Indiv. Diff. 26, 627–639. 10.1016/S0191-8869(98)00159-7

[B31] Hendricks J. Cutler S. J. (2004). Volunteerism and socioemotional selectivity in later life. J. Gerontol. B Psychol. Sci. Soc. Sci. 59, S251–S257. 10.1093/geronb/59.5.S251 15358799

[B32] Hess T. M. (2005). Memory and Aging in Context. Psychol. Bull. 131, 383–406. 10.1037/0033-2909.131.3.383 15869334

[B33] Hewig J. Hagemann D. Seifert J. Gollwitzer M. Naumann E. Bartussek D. (2005). A revised film set for the induction of basic emotions. Cogn. Emotion 19, 1095–1109. 10.1080/02699930541000084

[B34] Isaacowitz D. M. Wadlinger H. A. Goren D. Wilson H. R. (2006). Is there an age-related positivity effect in visual attention? A comparison of two methodologies. Emotion 6, 511–516. 10.1037/1528-3542.6.3.511 16938091

[B35] Jenkins L. M. Andrewes D. G. (2012). A new set of standardised verbal and nonverbal contemporary film stimuli for the elicitation of emotions. Brain Impair. 13, 212–227. 10.1017/BrImp.2012.18

[B36] John O. P. Gross J. J. (2007). Individual Differences in Emotion Regulation, in Handbook of Emotion Regulation, ed. J. J. Gross (New York, NY: Guilford Press), 351–372.

[B37] Keil A. Freund A. M. (2009). Changes in the sensitivity to appetitive and aversive arousal across adulthood. Psychol. Aging 24, 668–680. 10.1037/a0016969 19739923

[B38] Kendall C. P. Hollon S. D. Beck A. Hammen C. Ingram R. E. (1987). Issues and recommendations regarding use of the Beck Depression Inventory. Cognit. Ther. Res. 11, 289–299. 10.1007/BF01186280

[B39] Kennedy Q. Mather M. Carstensen L. L. (2004). The role of motivation in the age-related positivity effect in autobiographical memory. Psychol. Sci. 15, 208–214. 10.1111/j.0956-7976.2004.01503011.x 15016294

[B40] Kliegel M. Jäger T. Phillips L. H. (2007). Emotional development across adulthood: differential age-related emotional reactivity and emotion regulation in a negative mood induction procedure. Int. J. Aging Hum. Dev. 64, 217–244. 10.2190/U48Q-0063-3318-1175 17503687

[B41] Kunzmann U. Gruhn D. (2005). Age differences in emotional reactivity: the sample case of sadness. Psychol. Aging 20, 47–59. 10.1037/0882-7974.20.1.47 15769213

[B42] Labouvie-Vief G. (2003). Dynamic integration. Curr. Dir. Psychol. Sci. 12, 201–206. 10.1046/j.0963-7214.2003.01262.x

[B43] Larcom M. J. Isaacowitz D. M. (2009). Rapid emotion regulation after mood induction: age and individual differences. J. Gerontol. B Psychol. Sci. Soc. Sci. 64B, 733–741. 10.1093/geronb/gbp077 19808810 PMC2763016

[B44] Lawton M. P. Kleban M. H. Rajagopal D. Dean J. (1992). Dimensions of affective experience in three age groups. Psychol. Aging 7, 171–184. 10.1037/0882-7974.7.2.171 1610505

[B45] Leclerc C. M. Hess T. M. (2007). Age differences in the bases for social judgments: tests of a social expertise perspective. Exp. Aging Res. 33, 95–120. 10.1080/03610730601006446 17132566

[B46] Ledoux P. Besson L. (1994). Leon. Neuilly-sur-Seine: Columbia Pictures Gaumont Film Company.

[B47] Lench H. C. Flores S. A. Bench S. W. (2011). Discrete emotions predict changes in cognition, judgment, experience, behavior, and physiology: a meta-analysis of experimental emotion elicitations. Psychol. Bull. 137, 834–855. 10.1037/a0024244 21766999

[B48] Mammarella N. Di Domenico A. Palumbo R. Fairfield B. (2017). Self-generation and positivity effects following transcranial random noise stimulation in medial prefrontal cortex: a reality monitoring task in older adults. Cortex 91, 186–196. 10.1016/j.cortex.2016.11.005 27912894

[B49] Mather M. (2012). The emotion paradox in the aging brain. Ann. N.Y. Acad. Sci. 1251, 33–49. 10.1111/j.1749-6632.2012.06471.x 22409159 PMC3395773

[B50] Mather M. (2016). The affective neuroscience of aging. Annu. Rev. Psychol. 67, 213–238. 10.1146/annurev-psych-122414-033540 26436717 PMC5780182

[B51] Mather M. Carstensen L. L. (2005). Aging and motivated cognition: the positivity effect in attention and memory. Trends Cogn. Sci. 9, 496–502. 10.1016/j.tics.2005.08.005 16154382

[B52] Nowlan J. S. Wuthrich V. M. Rapee R. M. (2015). Positive reappraisal in older adults: a systematic literature review, Aging & Mental Health, 19, 475–484. 10.1080/13607863.2014.954528 25204368

[B53] Philippot P. (1993). Inducing and assessing differentiated emotion-feeling states in the laboratory. Cogn. Emot. 7, 171–193. 10.1080/02699939308409183 27102736

[B54] Piazza J. R. Charles S. T. Stawski R. S. Almeida D. M. (2013). Age and the association between negative affective states and diurnal cortisol. Psychol. Aging 28, 47–56. 10.1037/a0029983 23088196 PMC3609945

[B55] Reed A. E. Chan L. Mikels J. A. (2014). Meta-analysis of the age-related positivity effect: age differences in preferences for positive over negative information. Psychol. Aging 29, 1–15. 10.1037/a0035194 24660792

[B56] Rottenberg J. Ray R. D. Gross J. J. (2007). Emotion elicitation using films, in Handbook of Emotion Elicitation and Assessment, ed Allen J. A. C. J. J. B. (New York, NY: Oxford University Press), 9–28.

[B57] Russell J. A. (1980). A circumplex model of affect. J. Pers. Soc. Psychol. 39, 1161–1178. 10.1037/h0077714

[B58] Russell J. A. Barrett L. F. (1999). Core affect, prototypical emotional episodes, and other things called emotion: dissecting the elephant. J. Pers. Soc. Psychol. 76, 805–819. 10.1037/0022-3514.76.5.805 10353204

[B59] Samson A. C. Kreibig S. D. Soderstrom B. Wade A. A. Gross J. J. (2016). Eliciting positive, negative and mixed emotional states: a film library for affective scientists. Cogn. Emotion 30, 827–856. 10.1080/02699931.2015.1031089 25929696

[B60] Schaefer A. Nils F. Sanchez X. Philippot P. (2010). Assessing the effectiveness of a large database of emotion-eliciting films: a new tool for emotion researchers. Cogn. Emot. 24, 1153–1172. 10.1080/02699930903274322

[B61] Scheibe S. Blanchard-Fields F. (2009). Effects of regulating emotions on cognitive performance: what is costly for young adults is not so costly for older adults. Psychol. Aging 24, 217–223. 10.1037/a0013807 19290754 PMC2658623

[B62] Scheibe S. Carstensen L. L. (2010). Emotional Aging: recent Findings and Future Trends. J. Gerontol. B Psychol. Sci. Soc. Sci. 65B, 135–144. 10.1093/geronb/gbp132 20054013 PMC2821944

[B63] Streubel B. Kunzmann U. (2011). Age differences in emotional reactions: arousal and age-relevance count. Psychol. Aging 26, 966–978. 10.1037/a0023424 21517185

[B64] Watson D. Clark L. A. Tellegen A. (1988). Development and validation of brief measures of positive and negative affect: the PANAS scales. J. Pers. Soc. Psychol. 54, 1063–1070. 10.1037/0022-3514.54.6.1063 3397865

[B65] Weiner B. (1982). The emotional consequences of causal attributions, in Affect and Cognition, eds Clark M. S. Fiske S. T. (Hillsdale, NJ: L.E.A)

[B66] Westermann R. Spies K. Stahl G. Hesse F. W. (1996). Relative effectiveness and validity of mood induction procedures: a meta-analysis. Eur. J. Soc. Psychol. 26, 557–580. 10.1002/(SICI)1099-0992(199607)

